# Effect of Refractive Index of Substrate on Fabrication and Optical Properties of Hybrid Au-Ag Triangular Nanoparticle Arrays

**DOI:** 10.3390/ma8052688

**Published:** 2015-05-19

**Authors:** Jing Liu, Yushan Chen, Haoyuan Cai, Xiaoyi Chen, Changwei Li, Cheng-Fu Yang

**Affiliations:** 1 School of Information Engineering, Jimei University, Xiamen 361021, China; E-Mails: jingliu@jmu.edu.cn (J.L.); chenys@jmu.edu.cn (Y.C.); haoyuancai1@gmail.com (H.C.); 2 China–Australia Joint Laboratory for Functional Nanomaterials, Xiamen University, Xiamen 361005, China; E-Mail: lichangweichangwei@gmail.com; 3 Department of Physics, National University of Singapore, Singapore 117551, Singapore; E-Mail: a0123706@u.nus.edu; 4 Department of Chemical and Materials Engineering, National University of Kaohsiung, No. 700, Kaohsiung University Rd., Nan-Tzu District, Kaohsiung 811, Taiwan

**Keywords:** discrete dipole approximation (DDA), nanosphere lithography (NSL), hybrid Au-Ag nanoparticles, substrate refractive index

## Abstract

In this study, the nanosphere lithography (NSL) method was used to fabricate hybrid Au-Ag triangular periodic nanoparticle arrays. The Au-Ag triangular periodic arrays were grown on different substrates, and the effect of the refractive index of substrates on fabrication and optical properties was systematically investigated. At first, the optical spectrum was simulated by the discrete dipole approximation (DDA) numerical method as a function of refractive indexes of substrates and mediums. Simulation results showed that as the substrates had the refractive indexes of 1.43 (quartz) and 1.68 (SF5 glass), the nanoparticle arrays would have better refractive index sensitivity (RIS) and figure of merit (FOM). Simulation results also showed that the peak wavelength of the extinction spectra had a red shift when the medium’s refractive index *n* increased. The experimental results also demonstrated that when refractive indexes of substrates were 1.43 and 1.68, the nanoparticle arrays and substrate had better adhesive ability. Meanwhile, we found the nanoparticles formed a large-scale monolayer array with the hexagonally close-packed structure. Finally, the hybrid Au-Ag triangular nanoparticle arrays were fabricated on quartz and SF5 glass substrates and their experiment extinction spectra were compared with the simulated results.

## 1. Introduction

Surface plasmon resonances (SPRs) are surface electromagnetic waves that propagate in a direction parallel to the metal/dielectric (or metal/vacuum) interface. SPRs are used as the basis of many standard tools for measuring adsorption of material onto planar metal (typically gold and silver) surfaces or onto the surface of metal nanoparticles. However, in their simplest form, SPRs’ reflectivity measurements can be used to detect molecular adsorption, such as polymers, DNA, proteins, *etc.* [1]. In a simple situation, such as that of nearly monodisperse spherical gold nanoparticle arrays in solution, the extinction spectrum exhibits a single peak known as the localized SPRs (LSPRs) [2]. LSPRs are collective electron charge oscillations in metallic plane or nanoparticle arrays that are excited by light and LSPRs exhibit enhanced near-field amplitude at the resonance wavelength. For that, LSPRs’ spectroscopy of metallic nanoparticle arrays is a powerful technique for chemical and biological applications and different lab-on-a-chip sensors [3,4].

The discrete dipole approximation (DDA) is a method being used to compute scattering of radiation for particles having arbitrary shapes and having periodic structures [5]. The basic idea of the DDA algorithm was introduced in 1964 by DeVoe [6] who applied it to study the optical properties of molecular aggregation. Given a target of arbitrary geometry, one can calculate its scattering and absorption properties with the DDA method. Exact solutions to Maxwell’s equations of LSPRs are known only for special geometries such as spheres, spheroids, or cylinders, so approximate methods are generally required. However, the DDA method employs no physical approximations and can produce accurately enough results, which can give sufficient computer power to simulate the LSPRs optical properties of nanoparticle arrays.

In 1909, Lorentz [7] showed that the dielectric and refractive properties of a substance could be directly related to the polarizabilities of the individual atoms of which it was composed. With a particularly simple and exact relationship, the Lorentz–Lorenz equation, also known as the Clausius–Mossotti relation and Maxwell’s formula, the refractive index of a substance is related to its polarizability. Typical metals that support surface plasmons in the nanoparticle arrays’ structure are silver (Ag) [8], gold (Au) [9], and chromium (Cr) nanoparticle arrays [10] in single-layer structure and hybrid Ag-Au [5] and hybrid Ni-Au [11] nanoparticle arrays in bi-layer structure. In addition, Cr can be used as the interlayer [5,12] to improve the adhesive effect of nanoparticle arrays.

As we know, the refractive index sensitivity (RIS) and figure of merit (FOM value, defined as the ratio of RIS/FWHM (full width at half maximum)) of the LSPR sensors [13,14] are sensitive to the used substrates. In the past, the electron-beam lithography (EBL) [15] and photolithography [16] methods can be used to fabricate the nanoparticle arrays for LSPRs’ structures. Nanosphere lithography (NSL) [17,18] is one of the most low-cost and high-efficiency methods for producing periodically and geometrically tunable nanostructure arrays. In this paper, at first, the DDA method was used to simulate and find the RIS and FOM values of the hybrid nanostructure arrays on glasses with different refractive indexes. After finding glass refractive indexes had the better RIS and FOM values, the periodically hybrid Au-Ag triangular nanoparticle arrays were systematically grown on the glasses. The NSL method was also used to grow the hybrid Au-Ag triangular nanoparticle arrays. One important reason for using hybrid Au-Ag nanoparticle is that the Au can avoid Ag being oxidized or sulfurized. Glasses with different refractive indexes were used as the substrates, which were used to investigate the effect of different refractive indexes of the substrates on the optical properties of the hybrid Au-Ag triangular nanoparticle arrays.

## 2. Model Construction and Simulation

**Figure 1:**
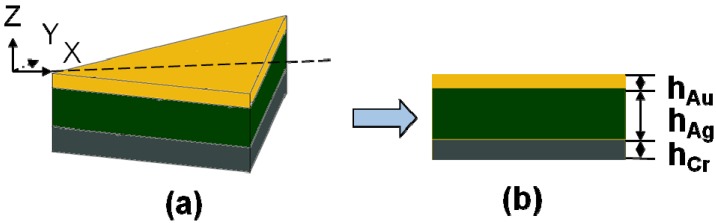
Schematic view of a single hybrid Au-Ag nanoparticle (**a**) In 3D view (**b**) Side cross-section view.

An implementation of electrodynamic theory called the discrete dipole approximation (DDA) can be used to model the experimentally measured extinction spectra. The DDA provides a convenient method for describing light scattering from nanoparticles or nanoparticle arrays of arbitrary shapes. Using the DDA algorithm, we could design and calculate the extinction spectra, RIS value, and FOM value of the hybrid Au-Ag triangular nanostructure arrays on different substrates. In the past, we had found that that the RIS value of the hybrid Au-Ag triangular nanoparticle arrays increased with the thickness of Cr interlayer from 4 to 12 nm and then decreased with further increase of Cr interlayer thickness to 20 nm [5]. Even the FOM value generally decreased with the increasing thickness of Cr thickness, we chose a suitable Cr interlayer thickness of 8 nm to improve the adhesion between Ag film and the substrates [5]. The corresponding schematic illustration of the hybrid nanoprism is shown in Figure 1: the structure was equilateral triangle with a side length of 180 nm. The thicknesses of Cr, Ag, and Au thin films were fixed on *h*_Cr_ = 8 nm, *h*_Ag_ = 35 nm, and *h*_Au_ = 5 nm, where *h*_Au_, *h*_Ag_, and *h*_Cr_ were defined in Figure 1b. The used substrates were quartz (with refractive index of 1.43), BAK1 glass (1.57), SF5 glass (1.68), SF10 glass (1.74), and SF6 glass (1.80), respectively.

**Figure 2:**
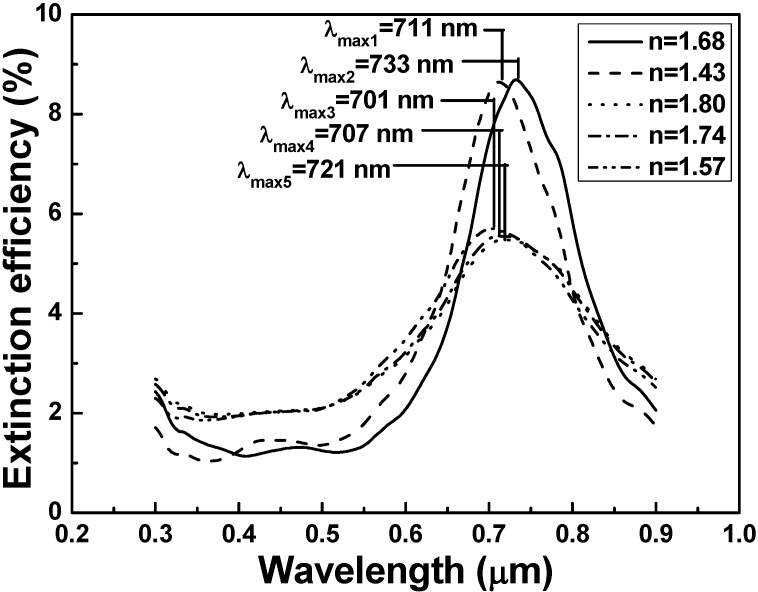
The extinction spectra results of compound nanostructure arrays as a function of substrate with different refractive indexes.

Extinction efficiency is a parameter used in physics to describe the absorption and scattering of electromagnetic radiation. Any changes in the parameters of metal nanoparticles or nanoparticle arrays could lead to the optical drift in extinction spectrum and thus influence the optical applications in practice. As Figure 2 shows, the peak value of the LSPR wavelengths had no apparent change but the peak intensity had large variation as the substrates’ refractive index was changed. However, the extinction intensity did not gradually change with gradual change in refractive index of the substrate and the higher extinction intensities were revealed in using quartz and SF5 glass as the substrates. SPR is the resonant oscillation of conduction electrons at the interface between a negative and positive permittivity material stimulated by incident light. Surface plasmon polaritons are surface electromagnetic waves that propagate in a direction parallel to the metal/dielectric and/or metal/vacuum interfaces. The resonance condition is established when the frequency of incident photons matches the natural frequency of surface electrons oscillating against the restoring force of positive nuclei. Since the wave is on the boundary of the metal and the external medium (air in this paper), these oscillations are very sensitive to any change in this boundary, such as the adsorption of molecules to the metal surface. The peak value of LSPR wavelengths had no apparent change because the peak value is mainly caused by the Ag film, which will be proven later. When quartz and SF5 glass are used as substrates, their maximum extinction efficiencies are higher than the values using other glasses as substrates. We believe that as the refractive indexes of 1.43 (quartz) and 1.68 (SF5 glass) are used as the substrates, the frequency of incident photons will match the natural frequency of surface electrons on the two substrates. For that, when the quartz and SF5 glass are used as the substrates, the extinction intensities will be higher.

In addition, as the quartz and SF5 glass were used as the substrates, their FWHM value of the extinction efficiency spectra were smaller than the FWHM values of the extinction efficiency spectra using other glasses as substrates. In this study, the main extinction peak of the hybrid Au-Ag triangular nanoparticle arrays was observed at around 700 nm. In our previous work [19], we had found that as only silver film was deposited as the nanoparticle arrays, the resonance peak of the extinction spectrum was around 700 nm. If the gold film was deposited on the surface of silver film to form the hybrid Au-Ag films, as the thickness of gold film in the hybrid Au-Ag nanoparticle arrays increased, the corresponding resonance extinction peak of the extinction spectrum had a blue shift. However, the hybrid nanoparticle arrays are composed of gold and silver films and the thickness of gold film is only 5 nm, which is very thin. For that, the silver film is the mainly sensitive medium and this result suggests that the main extinction peak around 700 nm is mainly caused by Ag plasmon.

The extinction efficiency spectra in Figure 2 show that the substrates with refractive indexes of 1.43 and 1.68 had the larger peak values. Two reasons will cause those results. Following the pure dephasing processes, an electron lattice equilibrium state is reached by transferring the electronic energy to the lattice through electron-phonon interaction on a subpico-second to several pico-second time scale. The energy is finally transferred to the environment by phonon-phonon interaction, which is dependent on the thermal conductivity and heat capacity of the medium and the coupling between the nanoparticle arrays and the surrounding mediums [20]. At first, when the quartz (refractive index 1.43) and SF5 (1.68) are used as substrates, high density grain boundaries with dense, high-frequency molecular type vibrations are present, which are effective in removing the energy of the excited electrons in the nanoparticle arrays and in transforming the energy of incident light into the phonon and thermal energy. Then, the ionic plasmas can absorb more resonance energy. Second, when the quartz and SF5 are used as substrates, the coupling effect between substrates and metal nanoparticle arrays will be enhanced, and the enhancement between photo fields will also enhance the local filed.

**Figure 3:**
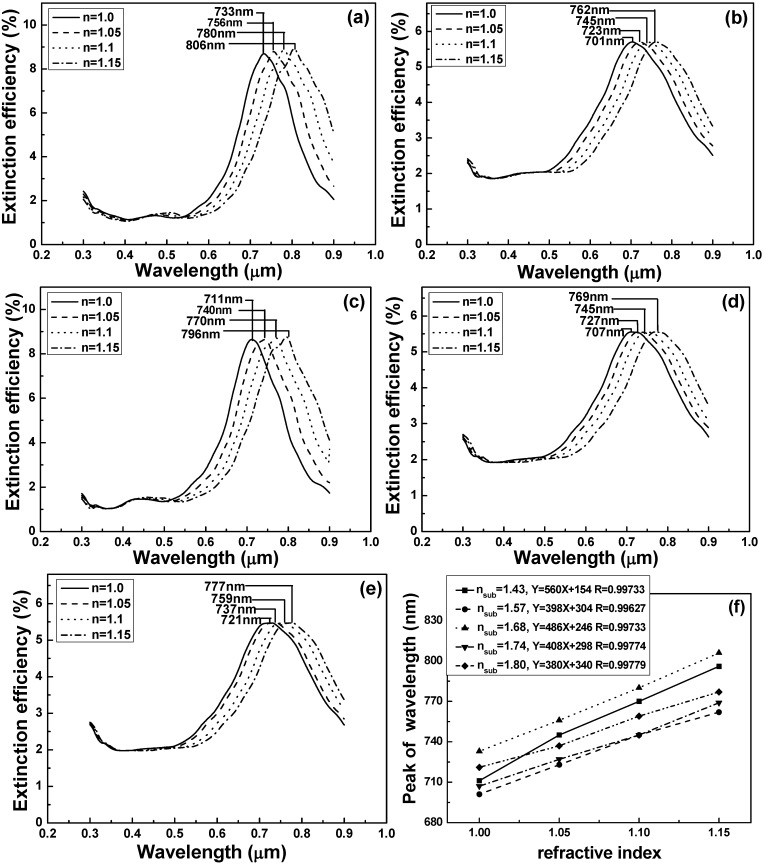
Effect of the substrates’ refractive indexes on the sensitivity of the hybrid nanostructure arrays. Extinction spectra in different media for substrate with refractive index of (**a**) 1.43; (**b**) 1.57; (**c**) 1.68; (**d**) 1.74; and (**e**) 1.80; and (**f**) for refractive index sensitivity curves.

Because the substrates with refractive index of 1.43 (quartz) and 1.68 (SF5 glass) had the higher extinction efficiency, as Figure 2 shows, they were used to investigate the effect of the substrates’ refractive indexes on the sensitivity of the hybrid Au-Ag nanostructure arrays. For that, the extinction efficiency spectra of the effective refractive index of the medium surrounding the nanostructure arrays were calculated. The refractive index sensitivity (RIS) is defined as *m* = Δλ/Δ*n* [5], where Δλ denotes the change in peak value of the wavelength of the extinction efficiency spectra and Δ*n* denotes the change in refractive index, respectively. As Figure 3a shows, as the substrate’s refractive index was 1.43 and the medium’s refractive index was increased from 1.0 to 1.15, the FWHM value and peak value of the extinction spectra had no apparent changes, the wavelength to reveal the peak value of the extinction spectra was shifted from 733 to 806 nm; however, as the substrate’s refractive index was 1.68 and the medium refractive index was increased from 1.0 to 1.15, the FWHM value and peak value of the extinction spectra also had no apparent changes, the wavelength to reveal the peak value of the extinction spectra was shifted from 711 to 796 nm, respectively.

The results shown in Figure 3a–e suggest that the peak wavelength of the extinction spectra had a red shift when the medium’s refractive index *n* increased. Figure 3f shows the relationships between the peak wavelengths of the extinction efficiency spectra and the medium’s refractive indexes, which could be used to investigate the RIS values. As results in Figure 3a–e are compared, the extinction efficiencies of using quartz (1.43) and SF5 glass (1.68) as substrates are higher than those of using other glasses as substrates. In addition, the FWHM values of using quartz and SF5 glass as substrates are smaller than those using other glasses as substrates. As the substrates’ refractive indexes are 1.43, 1.57, 1.68, 1.74 and 1.8, the RIS values of the hybrid Au-Ag triangular nanoparticle arrays are 560, 398, 486, 408, and 380 nm/RIU (refractive index unit), respectively. Because of having large extinction efficiencies and small FWHM values, only the FOM values of substrate’s refractive indexes of 1.43 and 1.68 were calculated, respectively, and the calculated values were 3.06 and 2.63, which are compared in Table 1.

**Table 1:** Comparisons of refractive index sensitivity (RIS), width at half maximum (FWHM), and figure of merit (FOM) using two different substrates.

Feature/Characteristic	*n*_substrate_ = 1.43	*n*_substrate_ = 1.68
RIS (nm/RIU)	560	486
FWHM (nm)	183	185
FOM	3.06	2.63

## 3. Fabrication of Hybrid Au-Ag Triangular Nanoparticle Arrays

**Figure 4:**
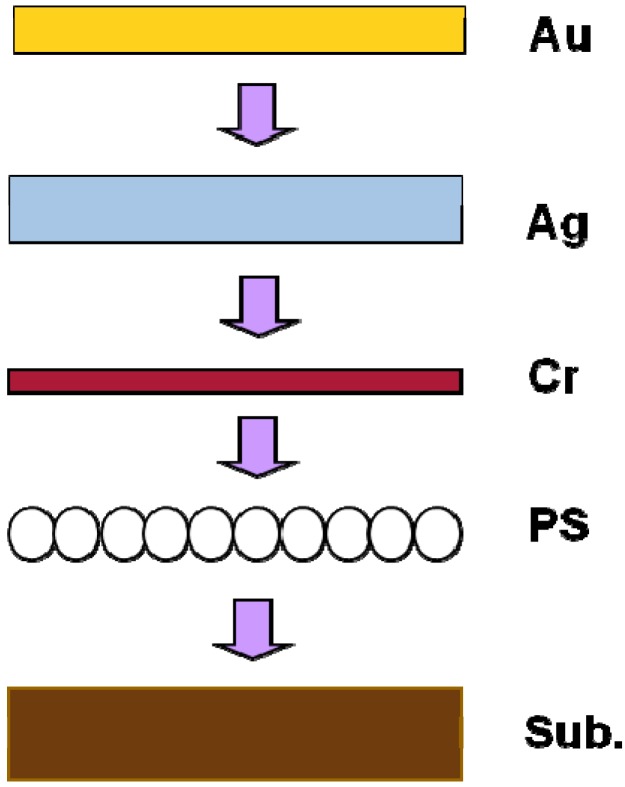
Schematic illustration for the fabrication process of the hybrid Au-Ag nanoparticle arrays.

In this study, the quartz and glass with different refractive indexes were used as substrates to study the effect of refractive index on the change of the LSPRs’ peak value and optical property of the hybrid Au-Ag nanoparticle arrays. Figure 4 illustrates the schematic view for the fabrication process of nanosphere lithography (NSL) method. In order to fabricate the hybrid nanoparticle arrays with the NSL method the polystyrene (PS) nanospheres were used as a deposition mask. The PS nanospheres with a mean diameter of 360 ± 10 nm and a concentration of 10 wt% in solution were purchased from Suzhou Nano-Micro Bio-Tech Co. Ltd. (Suzhou, China). The details for the preparation of the PS nanospheres were revealed in reference [5]. The depositions of Cr, Ag, and Au metals (all with 3N purity) were performed in a self-built thermal evaporator at a pressure of 5.0 × 10^−4^ Pa. The quartz and glass substrates were rotated at a speed of 16.5 rpm during the deposition process. The power for heating-up of the source materials was carefully increased in order to achieve homogeneous deposition. The deposition rate was about 4.0 nm/s for Cr thin film and the deposition rates were about 2.5 nm/s for both Au and Ag thin films, respectively. The thicknesses of the deposition thin films were monitored using a Dektak 3 Series surface profiler (Bruker, Billerica, MA, USA) to achieve an identical depth with a low reflectance. The deposition thicknesses of Cr, Ag, and Au thin films on PS nanospheres were about *h*_cr_ = 8 nm, *h*_Ag_ = 35 nm, and *h*_Au_ = 5 nm, respectively, by controlling the deposition time. After depositions of Cr, Ag, and Au thin films, the PS spheres were lifted off by immersing in absolute ethanol for about 5 s. The PS spheres were also removed by sonication (B3500S-MT, 140 W, 42 kHz, Branson, Danbury, CT, USA) in absolute ethanol to examine the adhesive ability of the hybrid Au-Ag nanoparticles on different substrates. The achieved PS mask and the structures of the achieved hybrid Au-Ag nanoparticle arrays on different substrates were characterized by scanning electron microscope (LEO-1530, Zeiss, Oberkochen, Germany). Ultraviolet visible (UV-Vis) spectra are obtained on a Cary 5000UV-Vis-NIR (175–3300 nm, Varian, New York, NY, USA) spectrophotometer.

## 4. Results and Discussion

**Figure 5:**
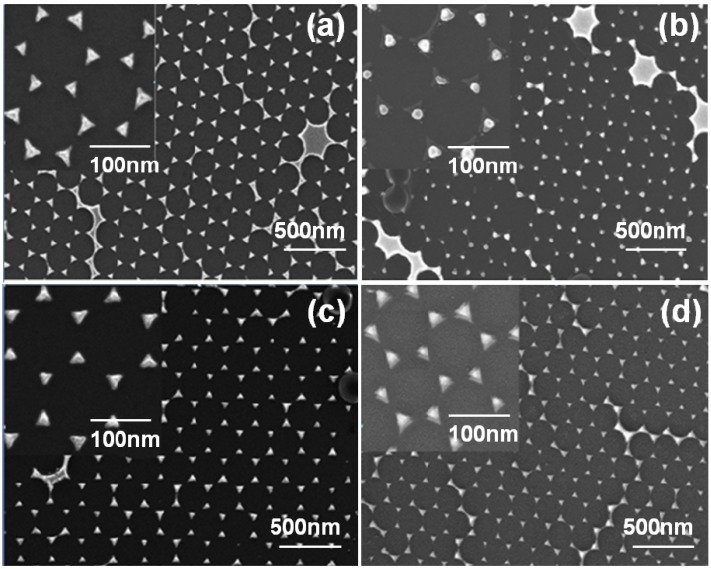
SEM (scanning electron microscope) images of the hybrid Au-Ag nanoparticle arrays deposited on two different substrates: (**a**) and (**b**): SF5, (**c**) and (**d**): quartz.

Figure 5 shows the top morphologies of the hybrid Au-Ag triangular nanoparticle arrays with the different substrates. Top morphologies of the deposited hybrid Au-Ag nanoparticle arrays, as Figure 5a,c show, exhibited a hexagonally arranged disc structure with triangular structures at the six corners. The hybrid Au-Ag nanoparticle arrays were regular and well-defined independent in the substrates, and no tiny cracks were observed in the structure. Those results suggest that the cohesive force between the PS nanospheres and the quartz and SF5 glass substrates seems to be strong enough. However, the results shown in Figure 5 revealed different results as the substrates were different. As SF5 glass substrates were used and the results in Figure 5a,b were compared, the as-deposited hybrid Au-Ag periodically nanoparticle arrays had a sharp angle in the triangular structure. The annealed Au-Ag periodically nanoparticle arrays were changed to quasi-half-ball structure and were shrunk together, the Cr interlayer was also observed.

The reason is caused by the deposition processes of Cr, Ag, and Au thin films, which are carried out at a higher temperature. Because of this, more defects will exist in the Cr thin film and interaction or collision mean free path between Cr and hybrid Au-Ag increases, and more Ag, Au atoms will be diffused into the Cr thin films. When the deposition processes of Ag and Au thin films are stopped, then the substrates will persist a quasi-quench process. According to thermodynamic theory, this process will cause the instability in the atoms’ stacking state. Therefore, from the dynamics theorem the Ag and Au have a lower separation speed, the quasi-quench process will keep the metal in the high-temperature state. For that, the Ag and Au can be uniformly deposited onto the Cr thin films. As the annealing duration persists for a period, the deposition multi-layer thin films will attain a condition of thermodynamic stability, which is changing from the non-equilibrium state to the equilibrium state. At this time, the separation condition will happen between Cr thin film and hybrid Au-Ag thin films. Then, the structure of Ag, Au thin films changes from triangle to quasi-half-ball because of the decrease of surface tension.

When the quartz was used substrates, the as-deposited hybrid Au-Ag periodically nanoparticle arrays had a sharp angle in the triangular structure, and the annealed nanoparticle arrays had no apparent change even the annealing process was used to treat on them, as Figure 5c,d were compared. As for the characteristics of the metal nanoparticles, the different morphologies after annealing can be attributed to that the Cr will diffuse into the SF5 glass substrate during the annealing process. As a result, the hybrid Au-Ag originally closed to the Cr will become adjacent to glass substrate. This phenomenon will lead to that the melting temperature of the Au-Ag nanoparticle arrays going lower than that of the Au-Ag-Cr nanoparticle arrays. However, the quartz substrate is a compact material, which can keep in stable even a higher temperature annealing process is treated, for that the Cr will not diffuse into the quartz substrate and the surfaces of the nanoparticle arrays are almost unchanged. 

**Figure 6:**
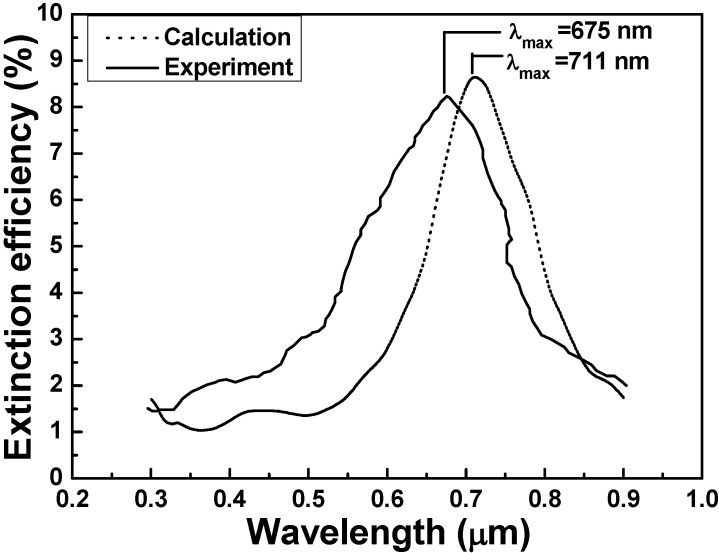
Extinction spectra of experiment and calculation with 1.43 refractive index substrate.

**Figure 7:**
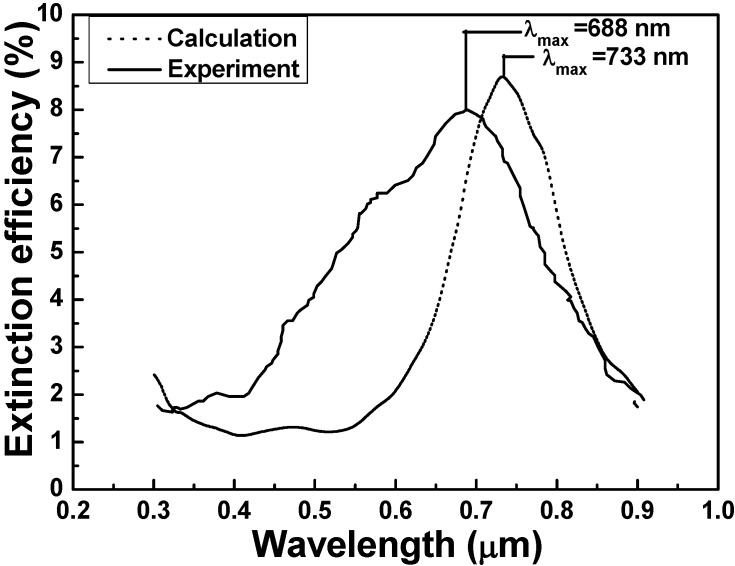
Extinction spectra of experiment and calculation with 1.68 refractive index substrate.

Figure 6 and Figure 7 compare the extinction spectra of the experimental results for *n*_medium_ = 1.0 with those of the simulated results extrapolated from the data in Figure 3a,b. For the results of the DDA calculation, the wavelength with the maximum extinction efficiency was 711 nm for substrate with refractive index of 1.68 and was 733 nm for substrate with refractive index of 1.43. Thus, for the measured results, the wavelength with the maximum extinction efficiency was 675 nm for substrate with refractive index of 1.68 and was 688 nm for substrate with refractive index of 1.43, respectively. Figure 6 and Figure 7 prove that the experimental results are generally in agreement with the calculated results. The major difference between with the calculation values and experiment values was that the wavelength with the maximum extinction efficiency had the blue shifts of 36 and 45 nm for using quartz and SF5 glass as substrates, respectively.

There are many reasons for the differences between the calculation values and experimental values, including the effects of different substrate [21] and the fabrication error caused by the uniformities of both size and shape of the hybrid Au-Ag nanoparticle arrays. In the DDA calculated model, the edge of the triangular nanoparticle arrays are straight while in real experimental results it will have a little warping and curving. Thus, effective medium theories can be proposed to explain the spectra of partially embedded objects [22], because the relative volumes of the two media have not been defined, except for the simple case of a half-embedded object. The exposed area and effective medium ideas are deficient because they fail to account for the change in LSPRs’ wavelength that arises from the asymmetric environment and distorting nanoparticle arrays. However, the comparisons of the calculated and simulated results of the 2D hexagonally arranged hybrid Au-Ag triangular nanoparticle arrays show that the DDA calculated model is suitable method for our experimental fabrication.

## 5. Conclusions

From the simulated results of the DDA numerical method, when the substrates’ refractive indexes were 1.43 (quartz) and 1.68 (SF5 glass), as the medium’s refractive index was increased from 1.0 to 1.15, the full width at half maximum of the LSPRs’ spectra and the wavelength to reveal the maximum extinction efficiency of the LSPRs’ spectra had no apparent changes. The wavelength to reveal the peak value of the LSPRs’ spectra was shifted from 733 to 806 nm for the substrate’s refractive index of 1.43 and was shifted from 711 to 796 nm for the substrate’s refractive index of 1.68, respectively. As the substrates’ refractive indexes were 1.68 and 1.43, the refractive index sensitivities of 486 and 560 nm/RIU were obtained in the hybrid Au-Ag triangular nanoparticle arrays. When the annealed process was used, the as-deposited hybrid Au-Ag periodically nanoparticle arrays on SF5 glass substrates were changed from a sharp angle in the triangular structure to quasi-half-ball structure and were shrunk together, and the Cr interlayer was also observed. The as-deposited hybrid Au-Ag periodically nanoparticle arrays on the quartz substrates had a sharp angle in the triangular structure and no apparent change even the annealing process was used to treat on them. For the results of the DDA calculation and substrates with refractive index of 1.68 and 1.43, the wavelengths to reveal the peak values of the LSPRs’ spectra were 711 and 733 nm. For the measured results and substrates with refractive index of 1.68 and 1.43, the wavelengths to reveal the peak values of the LSPRs’ spectra were 675 and 688 nm, respectively. However, the comparisons of the calculated and simulated results of the 2D hexagonally arranged hybrid Au-Ag triangular nanoparticle arrays show that the DDA calculated model is suitable to investigate the hybrid Au-Ag triangular nanoparticle arrays.
